# The Orthology Clause in the Next Generation Sequencing Era: Novel Reference Genes Identified by RNA-seq in Humans Improve Normalization of Neonatal Equine Ovary RT-qPCR Data

**DOI:** 10.1371/journal.pone.0142122

**Published:** 2015-11-04

**Authors:** Dragos Scarlet, Reinhard Ertl, Christine Aurich, Ralf Steinborn

**Affiliations:** 1 Centre for Artificial Insemination and Embryo Transfer, University of Veterinary Medicine, Vienna, Austria; 2 Genomics Core Facility, VetCore, University of Veterinary Medicine, Vienna, Austria; Texas Tech University Health Science Centers, UNITED STATES

## Abstract

**Background:**

Vertebrate evolution is accompanied by a substantial conservation of transcriptional programs with more than a third of unique orthologous genes showing constrained levels of expression. Moreover, there are genes and exons exhibiting excellent expression stability according to RNA-seq data across a panel of eighteen tissues including the ovary (Human Body Map 2.0).

**Results:**

We hypothesized that orthologs of these exons would also be highly uniformly expressed across neonatal ovaries of the horse, which would render them appropriate reference genes (RGs) for normalization of reverse transcription quantitative PCR (RT-qPCR) data in this context. The expression stability of eleven novel RGs (*C1orf43*, *CHMP2A*, *EMC7*, *GPI*, *PSMB2*, *PSMB4*, *RAB7A*, *REEP5*, *SNRPD3*, *VCP* and *VPS29*) was assessed by RT-qPCR in ovaries of seven neonatal fillies and compared to that of the expressed repetitive element *ERE-B*, two universal (*OAZ1* and *RPS29*) and four traditional RGs (*ACTB*, *GAPDH*, *UBB* and *B2M*). Expression stability analyzed with the software tool RefFinder top ranked the normalization factor constituted of the genes *SNRPD3* and *VCP*, a gene pair that is not co-expressed according to COEXPRESdb and GeneMANIA. The traditional RGs *GAPDH*, *B2M*, *ACTB* and *UBB* were only ranked 3rd and 12th to 14th, respectively.

**Conclusions:**

The functional diversity of the novel RGs likely facilitates expression studies over a wide range of physiological and pathological contexts related to the neonatal equine ovary. In addition, this study augments the potential for RT-qPCR-based profiling of human samples by introducing seven new human RG assays (*C1orf43*, *CHMP2A*, *EMC7*, *GPI*, *RAB7A*, *VPS29* and *UBB*).

## Introduction

In recent years the mare has intensively been used as an animal model for age-associated changes in human reproductive medicine [1]. Better approaches for the analysis of the molecular mechanisms behind ovarian development and maturation would help to prevent and treat infertility in both the human and the horse. The developing fetal equine ovary possesses several morphologic and developmental differences compared to other domestic species [2]. After the interstitial cell hypertrophy and hyperplasia that results in marked gonadal enlargement during the time spent in utero, a period of regression and ovarian cortical migration follows [2]. Recently, we have described the presence of tertiary follicles up to 4 mm in diameter in newborn fillies [3]. However, the regulatory mechanisms behind these processes have not been elucidated yet.

Quantitative real-time RT-PCR (RT-qPCR) is the most commonly used method for measuring transcript copies being especially useful when samples contain small numbers of cells [4]. The accuracy of RT-qPCR measurement is strongly dependent on appropriate normalization—usually accomplished using reference genes (RGs; [5]). RGs are mainly derived from the class of housekeeping genes required for the maintenance of basal cellular functions that are essential for the existence of a cell, regardless of its specific role in the tissue or organism [6]. RGs are the major drawback for RT-qPCR, as their validation on an individual basis for all treatment and experimental conditions is essential for accurate data interpretation, especially when normalizing for inter-sample variability [7]. Depending on the biological context, two [8,9] or three [8,10,11] of the most stably expressed RGs are sufficient to normalize the expression.

Over the past years, several mammalian genes have been intensively used as internal controls in gene expression studies, such as the traditional RGs *RN18S* (*18S rRNA*), *GAPDH*, *ACTB*, *HPRT*, *B2M* and *ALB*. Some of the latter genes (*ACTB*, *GAPDH* and *RN18S*) appear in 70% of the publications on RNA based qPCR as the solely normalizer, a fact that makes it highly unlikely that these have been validated [12]. Traditional RGs were derived by educated guesswork in the era of Northern blotting [13–15]. In the absence of any transcriptome-wide expression information, the expression uniformity of these genes was supposed to be stably expressed not only in model species, but also in their genetic relatives and hence frequently used as normalizers across plants and animals [16–19]. Some years ago, the orthology clause was suggested for a particular target context, the liver of mice, cattle and pig [20]. The clause indicated a similar expression of gene orthologs across related species. A huge amount of information, including novel aspects on human and mouse transcriptomes, was recently uncovered through RNA-seq [6,21]. This has opened a new dimension for studying mechanisms of molecular regulation. This data also indicated a substantial conservation of transcriptional programs between human and mouse by uncovering a distinct class of genes with levels of expression that have been constrained early in vertebrate evolution [22]. In detail, it was found that evolutionary constraint in gene expression levels is associated with conserved epigenetic marking and a characteristic post-transcriptional regulatory program.

Stability of RNA expression can be assessed by software tools such as GeNorm [23], NormFinder [24] or BestKeeper [25]. These major computational programs are integrated by the tool RefFinder ([26], http://fulxie.0fees.us/?type=reference), thus compensating for their individual weaknesses.

No gene exhibits a stable pattern of expression across all tissues and conditions of an individual [20]. Commonly used reference genes such as *ACTB*, *GAPDH*, *UBC* and *RN18S* can vary considerably depending on tissue types, developmental stage, sex, pathology, and experimental conditions [27]. For example, in whole blood donated by healthy human individuals, expression of *ACTB*, *GAPDH* or *HPRT* differed by a factor of 20- to 25-fold [28]. This level of variation considerably exceeds the strong threshold criterion of Δ*Cq* ≤ ±0.5 (twofold expression alteration) recommended for RG suitability [7]. Only few genes, termed “universal” reference genes, are relatively uniformly expressed across a wide range of conditions in men or mice [20]. For each biological context, a subset of genes exists that is more stably expressed than traditional normalizers or RGs that were selected by meta-analysis based on their expression stability across a plenitude of experimental conditions [20].

So far, there is a lack of reliable RGs for the biological context of this study—the neonatal ovary of *Equus caballus*. Here we tested the application of the orthology clause in the next generation sequencing era for this experimental setup. We targeted the equine orthologous genes of the most stably expressed human counterparts identified by RNA-seq from a wide panel of human tissues (Illumina Human Body Map 2.0) including the ovary. We argued that the extended number of transcripts identified, a resolution down to the level of individual exons and the expression consensus obtained by profiling across a large set of target tissues would facilitate a successful application of the clause, hence improve the gene repertoire for normalization of RT-qPCR data in this context.

## Material and Methods

In agreement with University Guidelines for Good Scientific practice, owners’ approval for scientific use of post-mortem material was obtained. In accordance with Austrian legislation on animal experimentation, collection of postmortem material from animal that had to be euthanized for veterinary medical reasons is not considered animal experimentation. Thus neither an animal experimentation license issued by the competent authority nor an internal approval by the University Animal Care and Use Committee was required.

### Biological material

Fourteen ovaries were collected from euthanized newborn female foals (*n* = 7). Euthanasia was performed due to various pathological conditions not related to the female genital tract using 10 mg/kg Thiopental (Inresa, Freiburg, Germany) followed by 4–6 ml/50kg T61 (MSD Animal Health, Vienna, Austria). The average age of foals was two days (range: 0 to 8 days).

### Sample preparation and RNA extraction

**Fig 1:**
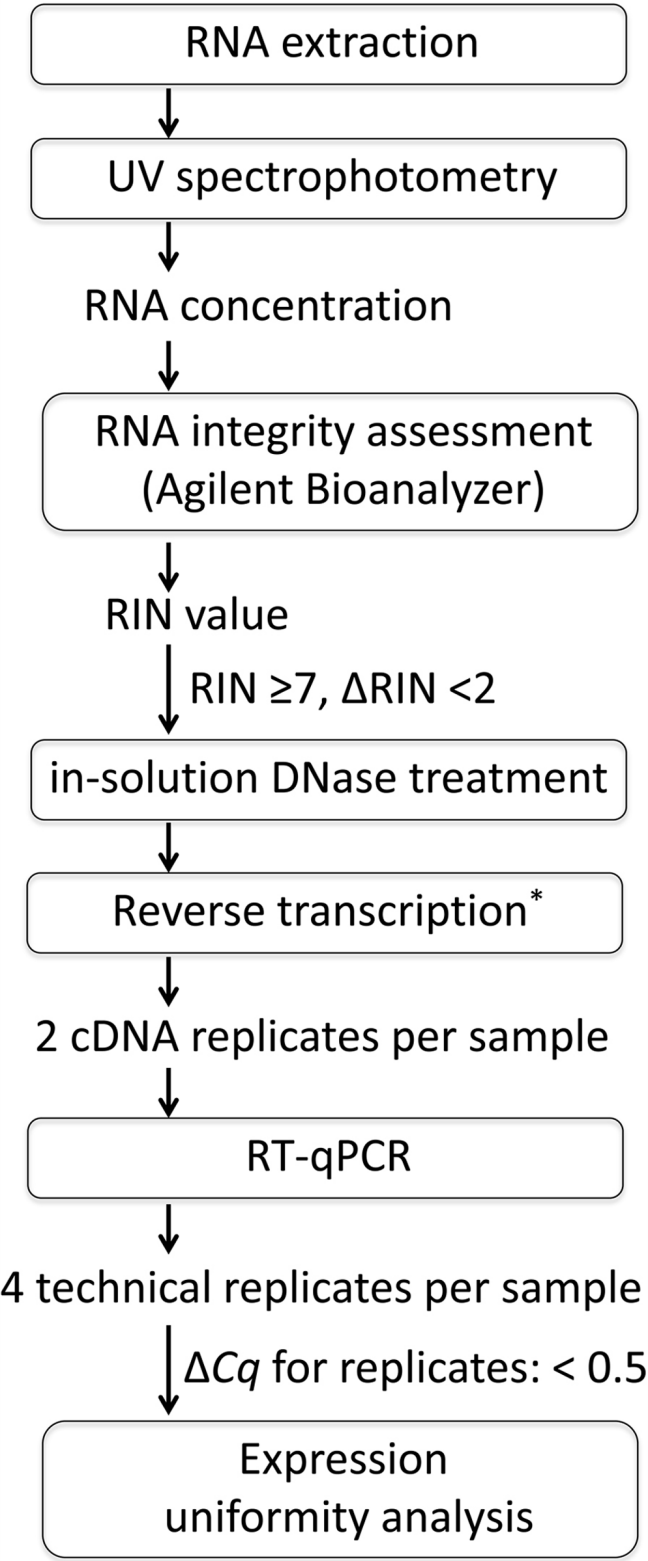
Processing of RNA for expression uniformity analysis by RT-qPCR. *Input RNA might contain nucleotides or short DNA fragments as a result of DNase digestion of co-purified DNA. The same amount of RNA was used for RT as concluded from a second spectrophotometric measurement of RNA mass before the RT step and based on the subsequent determination of *Cq* values for the rRNA genes *RN18S* and *RN28S* (Fig 2).

Fresh ovary biopsies were embedded in Tissue-Tek O.C.T. compound (Sakura Finetek, Alphen aan den Rijn, Netherlands), snap-frozen in liquid nitrogen and then stored at -80°C for less than 12 months. Frozen sections of 5 × 100 μm tissue were prepared on the cryostat CryoStar NX70 (Thermo Scientific, Waltham, USA). After removal of excess embedding medium, the sections were placed into 2 ml-screw caps filled with 1.3 mm ceramic beads (Peqlab Biotechnologie GmbH, Erlangen, Germany) and 350 μl RLT lysis buffer supplemented with 3.5 μl β-mercaptoethanol (Qiagen, Hilden, Germany). Mechanical homogenization was performed on the MagNA Lyser instrument (Roche, Rotkreuz, Switzerland) at 6500 rpm for 20 s followed by cooling on ice for 1 min. If necessary, the homogenization step was repeated. Total RNA was extracted using the RNeasy Micro Kit (Qiagen) according to Qiagen’s RNeasy Micro Kit protocol for fibrous tissues except an extended time for Proteinase K incubation (30 min). The procedure included on-column DNase I digestion for 15 min to remove residual DNA that can generate a false positive signal in some of the RT-qPCR assays. RNA concentration was determined by UV spectrophotometry on the NanoDrop 2000c (Peqlab Biotechnologie GmbH). RNA integrity was assessed by capillary electrophoresis on the Agilent 2100 Bioanalyzer (Agilent Technologies, Santa Clara, USA) using the RNA 6000 Nano Kit (Agilent Technologies). Only samples with RNA integrity number (RIN) values of at least seven were used for expression analysis (Fig 1). To further reduce unspecific signals from the presence of genomic DNA as concluded from a RT-qPCR assay against a traditional RG, *ACTB* (data not shown), samples were again DNase digested (TURBO DNA-free Kit; Life Technologies, Austin, USA). Exemplarily, two of the seven samples were again assessed on the Agilent Bioanalyzer to exclude that the second DNase digestion had affected the integrity of the RNAs (RIN > 7, ΔRIN of 0 and 1.0).

### Selection of RG candidates and design of RT-qPCR primers

Eleven genes showing the most ubiquitous and uniform expression across the ovary and 15 other normal human tissue types [6] were selected as novel RG candidates, namely *C5H1orf43* (ortholog of human *C1orf43)*, *CHMP2A*, *EMC7*, *GPI*, *PSMB2*, *PSMB4*, *RAB7A*, *REEP5*, *SNRPD3*, *VCP*, and *VPS29*. This expression data aiming at housekeeping gene detection in the era of massive parallel sequencing and RNA-seq was part of the Human BodyMap 2.0 project (www.ebi.ac.uk/arrayexpress/experiments/E-MTAB-513). This RNA-seq data delivered expressions at the exon level. This measure takes care of normalization for the two most obvious factors affecting the raw number of reads per gene, transcript, or exon: the total number of reads produced and their length. The variation of exon expression given as reads per kilobase per million mapped reads (RPKM) is estimated by the standard deviation of log_2_ (RPKM) over samples, abbreviated as SD(log_2_(RPKM)).

DNA and cDNA sequences including the transcript isoforms available for the horse were retrieved from NCBI. Aligning them with the orthologous human sequences allowed identifying primer target sequences that were conserved across the two species and less affected by individual polymorphisms.

A biological sample is composed of different types of cells mixed at different proportions. If an RT-qPCR assay is sensitive to alternative splicing, sample heterogeneity can lead to the identification of artificially deregulated transcript isoforms and in case of a RG to an increased expression variation. Therefore, our protocol was designed to target exons that are present in multiple tissue types.

As the exon/intron architecture of genes influences the way of mRNA processing, it was argued that selecting flanking introns of sizes around 1 to 2 kb would be an appropriate compromise to reduce the chance of alternative splicing caused by exon skipping and the alternative usage of 5’ or 3’ splice sites (based on human data from [29]). In general, the orthologous human exons targeted by the equine RT-qPCR assays were moderately to highly and uniformly expressed across the panel of human reference tissues (SD(log_2_(RPKM)) of 0.32 to 0.52 [6]). In a single case (*SNRPD3*), a less stable exon (SD(log_2_(RPKM)) = 0.70 [6]) had to be accepted as a target region in order to achieve the proposed size (1 to 2 kb) of the flanked intron.

To avoid co-amplification of contaminating genomic DNA due to the short elongation time, a critical size limit of at least 750 bp was adopted for the central intron, *i*.*e*. the one flanked by the target exons in genomic DNA. The limit was met for nine of the eleven novel putative RGs (Table 1). Two of the primer pairs flanked short introns of 73 and 412 bp (*CHMP2A* and *PSMB4*, respectively). In these cases, the minus RT control routinely performed for each experimental RNA confirmed specificity of mRNA amplification.

**Table 1:** Details on qPCR assays.

Gene symbol	NCBI accession number	5’-3’ sequence	E	Amplicon size(bp)	Targeted exonsF, R	Central intronsize (bp)	Upstreamintron size(bp)	Down-streamintron size (bp)	Assay ID
***C5H1orf43***	*XM_003365015*.*2*,*XM_001496636*.*2*,*XM_001496656*.*2*,*XM_008526884*.*1*,*NW_007673359*.*1*	F:GCTATCAGGAGGCCCTGAGT R:GGAGGTATGTCACCTGGATGG	1.01	141	6,7	2020	87	-	Hs:8778
***CHMP2A***	*XM_005596626*.*1*,*XM_008544637*.*1*,*XM_008544638*.*1*	F:ATGGGCACCATGAACAGACAG R:TCTCCTCTTCATCTTCCTCATCAC	0.90	151	1,2	73	-	90	Hs:8779
***EMC7***	*XM_001503666*.*3*,*XM_008516349*.*1*	F:GTCAGACTGCCCTATCCTCTCC R:CATGTCAGGATCACTTGTGTTGAC	1.00	177	3,4	1302	3781	2214	Hs:8784
***GPI***	*XM_005596131*.*1*,*XM_008515114*.*1*,*XM_001490607*.*3*	F:CCAAGTCCAGGGGCGTG R:CTTGTTGACCTCTGGCATCACA	0.90	158	3,4	1433	358	4394	Hs:8780
***PSMB2***	*XM_001503676*.*3*, *XM_003364409*.*2*, *XM_008533360*.*1*	F:ATATCATGTCAACCTCCTCCTGG R:AAGCTCCACTGCCCTCTCAC	0.90	187	4,5	1475	18074	2003	
***PSMB4***	*XM_001492317*.*4*, *XM_005610132*.*1*, *XM_008515015*.*1*, *XM_005613704*.*1*	F:CTTGGTGTAGCCTATGAAGCCC R:CCAGAATTTCTCGCAGCAGAG	0.93	82	4,5	412	178	216	
***RAB7A***	*XM_001488301*.*3*, *XM_008506841*.*1*, *XM_008506842*.*1*	F:CTGGTATTTGATGTGACTGCCC R:AATCGTCTGGAACGCCTGC	1.02	252	3,4	1623	8255	4181	Hs:8782
***REEP5***	*XM_005599722*.*1*, *XM_008535732*.*1*	F:CCTGAAGCACGAGTCCCAG R:CCCAGTAAATTCACGGCAGC	0.92	114	3,4	5086	10372	-	
***SNRPD3***	*XM_001489060*.*4*, *XM_008511652*.*1*	F:ACGCACCTATGTTAAAGAGCATG R:CACGTCCCATTCCACGTC	0.99	120	3,4	1861	16047	-	
***VCP***	*XM_005605574*.*1*, *XM_008508520*.*1*	F:GAGTGAGATCAGGCGAGAACG R:CCTCTTCCACCTCCATGGC	0.92	56	14,15	1673	279	154	
***VPS29***	*XM_001495080*.*4*,*XM_001495099*.*4*,*XM_008508375*.*1*	F:AAACCTTTGCACCAAAGAGAGTTATG R:CAACAGTCACAACTTTCTGTTCTGG	0.90	119	2,3	2105	4766	244	Hs:8786
***ACTB***	*NM_001081838*.*1*	F:CGGGACCTGACGGACTA R:CCTTGATGTCACGCACGATT	0.91	94	3,3	-	448	85	
***GAPDH***	*NM_001163856*.*1*,*XM_008513936*.*1*	F:GGCAAGTTCCATGGCACAGT R:CACAACATATTCAGCACCAGCAT	0.90	129	4,5	119	82	127	
***UBB***	*AF506969*.*1*	F:TTCGTGAAGACCCTGACC R:CCTTATCCTGGATCTTGGC	0.92	91	2,2	-	NA	-	Hs:8781
***B2M***	*XM_005602594*.*1*,*XM_005602595*.*1*	F:CCTGCTCGGGCTACTCTC R:CATTCTCTGCTGGGTGACG	0.94	89	1,2	3266	-	583	
***OAZ1***	*CD467318*.*1*	F:CGGCTCCCTGTACATCGAGAT R:GCGGTTCTTGTGGAAGCAGAT	0.98	133	3,4	NA	NA	NA	
***RPS29***	*XM_001494060*.*4*,*XM_008528084*.*1*	F:CAGCTCTACTGGAGCCATC R:ACATGTTGAGGCCGTACTTC	0.96	103	1,2	207	-	1133	
***ERE-B***	-	F:GTGGCCTAGTGGTTAAGTTCG R:ACGCCGCCACAGCATG	0.93	101–113	intron-free gene				
***RN18S***	*NR_046271*.*1*	F:CCATTCGAACGTCTGCCCTA R:TCACCCGTGGTCACCATG	0.94	68	intron-free gene				
***RN28S***	*NR_046309*.*1*	F:TAGCCAAATGCCTCGTCATCT R:AACAGTTAGGGACAGTGGGAATCT	0.96	74	intron-free gene				

F and R: forward and reverse primers; *E*: amplification efficiency; NA: information not available due to incomplete sequence data.

Hs: *Homo sapiens*.

Assay ID according to RTPrimerDB (http://medgen.ugent.be/rtprimerdb/).

Each RT-qPCR assay targeted all of the two to four transcripts available at the NCBI Gene database for *E*. *caballus* and *E*. *przewalskii* (S1 File).

In order to exclude differences in the amount of co-isolated DNA, samples were analysed for expression of *18S* und *28S* ribosomal fractions.

For comparison four traditional RGs, two universal RGs and one expressed repetitive element were selected. The traditional RGs were selected just by educated guess-work and tradition (*ACTB* and *GAPDH*, [30]) or based on stable expression across a panel of equine tissues (*UBB* and *B2M*, [31,32]). The universal RGs, *OAZ1* and *RPS29*, exhibited highly uniform expression in men and mice across a plenitude of conditions [33–35]. The assay for the expressed repetitive element targeted a consensus sequence deduced from all equine repetitive element B (*ERE-B*) sequences available at the SINEBase database ([36]; http://sines.eimb.ru/).

Primers for the novel candidates, the universal RGs and the expressed repetitive element were designed in the Primer Express 2.0 program (Life Technologies, Foster City, USA). Primer dimerization was assessed by the NetPrimer web tool (Premier Biosoft, Palo Alto, USA) freely available at www.premierbiosoft.com. Amplicon specificity was evaluated by the Primer-BLAST tool of NCBI using the non-redundant database of the taxid “horses”. Amplicon folding was analyzed using the Mfold web server [37]. Primer sequences and assay details are listed in Table 1.

### RT-qPCR

In order to account for variations during the RT step, two replicates of each RNA sample were converted into cDNA using the High Capacity Reverse Transcription Kit (Life Technologies) (Fig 1). For each replicate, 2 μg total RNA input were reverse-transcribed with random hexamers in a reaction volume of 40 μl following the recommended protocol. Since sensitivity towards genomic DNA contamination differs greatly between assays [38,39], controls in which no reverse transcriptase was added were run for each assay and each experimental RNA. RT-qPCR was performed in duplicates for each cDNA replicate (equals four technical replicates for each RNA sample) using the Power SYBR Green Master Mix (Life Technologies). The 20-μl reaction included 150 nM of each primer and 2 μl of five-fold diluted cDNA. All qPCR runs were performed on a Viia7 Real-Time PCR System (Life Technologies). After an initial denaturation at 95°C for 10 min, 40 amplification cycles of 95°C for 15 s and 60°C for 1 min were performed. Amplicon dissociation was monitored over a temperature range of 60°C to 95°C. Additionally, PCR products were separated on a 2% agarose gel stained with GelGreen (Biotium, Hayward, USA) and visualized by blue light excitation (S2 File). Absence of unspecific amplification derived from the mastermix was confirmed by melting curve analysis of the no template control product. Absence of PCR inhibitors was assessed by RT-qPCR against *RN18S* using eightfold dilution of all experimental cDNAs as template.

Reverse transcription was repeated if the *Cq* values of two cDNA replicates differed by more than 0.5 cycles.

A standard curve was generated for each assay for calculating PCR efficiency (*E*) from the term 10^−1/s^—1, were *s* is the slope of the graph obtained by plotting the *Cq* value against the log_10_ of the cDNA input [40]. For *Cq* values generated at *E* < 1, efficiency corrected *Cq* values were obtained from the term *Cq* * *log*_10_(*E* + 1)/*log*_10_(2) [41].

### Data analysis

The efficiency-corrected *Cq* values were processed using the RefFinder tool integrating the currently available major computational programs geNorm, Normfinder, BestKeeper, and the comparative ΔΔ*Cq* method to compare and rank the tested candidate reference genes ([26]; http://fulxie.0fees.us/?type=reference). Based on the rankings from each program, RefFinder assigns an appropriate weight to an individual gene or set of genes and calculates the geometric mean of their weights for the overall final ranking. A normalization factor, *i*.*e*. the geometric mean of several top-ranking genes, was considered suitable as soon as it was ranked ahead of all single genes.

In order to exclude the association of highly coexpressed genes which could affect the stability of the normalization factor, coexpression was identified with COXPRESdb [42] software, which calculates Pearson’s correlation coefficient for each gene pair, and then transfers these values to the mutual rank value, which is the geometric average of asymmetric ranks in coexpressed gene lists.

To confirm COXPRESdb results, the predicted coexpression of the novel RGs was analysed with GeneMANIA [43], a software based on functional association data such as protein and genetic interactions, pathways, co-expression, co-localization, and protein domain similarity. The default approach, the automatically selected weighting method, was used when combining all networks into the final composite. In case of our search list with more than five genes, GeneMANIA assigns weights to maximize connectivity between all input genes using the ‘assigned based on query gene’ strategy. According to this strategy the weights are chosen automatically using linear regression, to make genes on the search list interact as much as possible with each other and as little as possible with genes not on this list.

As *E*. *caballus* species is neither reviewed by COEXPRESdb, nor by GeneMANIA, *Homo sapiens* databases were used in both cases.

The RT-qPCR data of this work comply with the Minimum Information for Publication of Quantitative Real-Time PCR Experiments (MIQE) guidelines [5].

### Statistical analysis

Statistical analysis was performed using SPSS Statistics 22 (IBM-SPSS, Armonk, NY, USA). The Kolmogorov–Smirnov test was used to demonstrate a normal distribution of efficiency corrected and log transformed *Cq* values of the candidate RGs. Correlations between parameters were analyzed using the Pearson’s correlation test. A *P* < 0.05 was considered significant.

## Results and Discussion

This study addressed the orthologous use of exons ubiquitously and stably expressed across a wide set of human tissues for improving RT-qPCR normalization in the context of the equine ovary. If genome-wide transcript data is missing for a specific biological context in a related species, for example the neonatal ovary of the horse, this assumption could be applied to select RGs for improving RT-qPCR data normalization.

Choosing the most suitable RGs for one experiment is being complicated by the fact that the majority of mRNAs undergoes alternative polyadenylation [44]. This is more likely to occur when the target exons are located in the three prime parts of a gene. In addition, most human multi-exon genes are known to be alternatively spliced. Out of these, 85% have a minor isoform frequency of at least 15% [45]. For the human genome, alternative splicing by exon skipping is more likely to occur when exons are flanked by long introns, whereas the activation of alternative splice sites is less likely if the flanking introns are long [29,46]. Therefore, for the first time we considered these crucial principles when selecting appropriate exons for targeting by the RT-qPCR primers.

The eleven novel RGs showed high to low expression levels (efficiency-corrected *Cq* values < 15 to > 30, respectively; S3 File) and span a wide range of biological functions in general (S4 File). According to the GeneMANIA prediction server, they are not part of the same biochemical process (“high” false discovery rate of 5.44e-2) with exception of *PSMB2*, *PSMB4* and *VCP* which belong to the proteasome complex.

**Fig 2:**
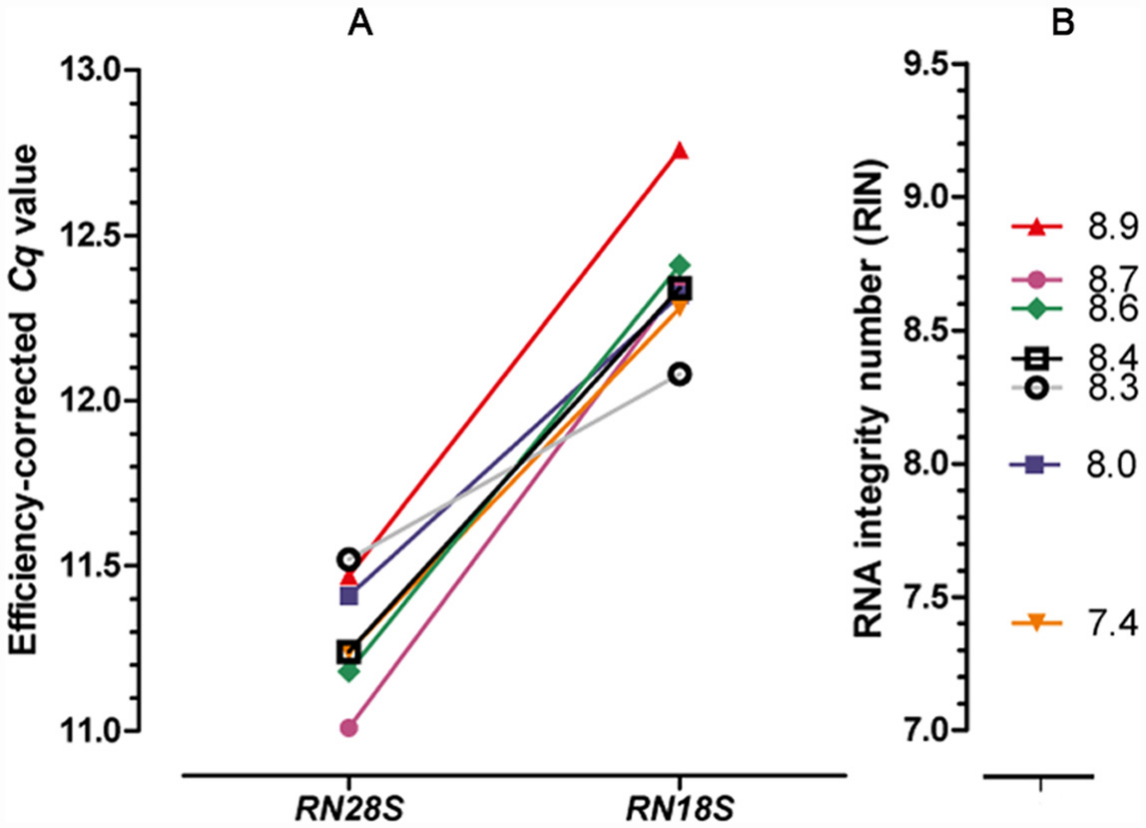
RT-qPCR profiling of the rRNA genes *RN18S* and *RN28S* (A) and assessment of RNA integrity (B).

Intactness of experimental RNAs was confirmed by RIN values above the required threshold of at least seven (range: 7.4 to 8.9; Fig 2). Co-purification of traces of DNA during RNA extraction is inevitable [39] with some RNA preparations yielding virtually pure RNA and others virtually pure DNA and this is independent of tissue type or operator [47]. Therefore, after determination of RIN values, samples were digested by DNase I to include also primer pairs from previous publications that were not intron-spanning or that flanked smaller introns (< 750 bp). Considering that in solution digestion did not remove the degradation products of carry-over DNA, we ruled out that the experimental cDNAs were derived from different proportions of total RNA due to differences in the amount of co-extracted genomic DNA. This was concluded from similar *Cq* values of RT-qPCRs targeting the main contributors to total RNA mass, *RN18S* and *RN28S* (Fig 2). In other words, the similarity of *Cq* values indicated that the selection of novel uniformly expressed genes for normalization of RT-qPCR data in the context of postnatal equine ovaries would not be affected by differences in the amount of co-isolated DNA. We argued that the approximately 50% change in the amount of input RNAs concluded from a Δ*Cq* value of approximately 0.5 for the *RN28S* transcript (Fig 2) did not affect the aim of this study—selection of novel RGs, since *Cq* values of *RN28S* and putative RGs were not correlated (*P* > 0.1).

Absence of PCR inhibitors from the experimental cDNAs was confirmed by RT-qPCR profiling of experimental cDNAs and their eightfold dilutions with an assay against *RN18S*. The low standard deviation of *Cq* values determined for the eightfold dilutions of the cDNAs indicated homogeneity regarding sample purity (mean Δ*Cq* of 2.7 ± 0.1 corresponding to *E* = 1). The slight deviation from the theoretical Δ*Cq* of three was attributed to inter-assay variation considering that the sets of diluted and undiluted samples were run on different plates.

**Fig 3:**
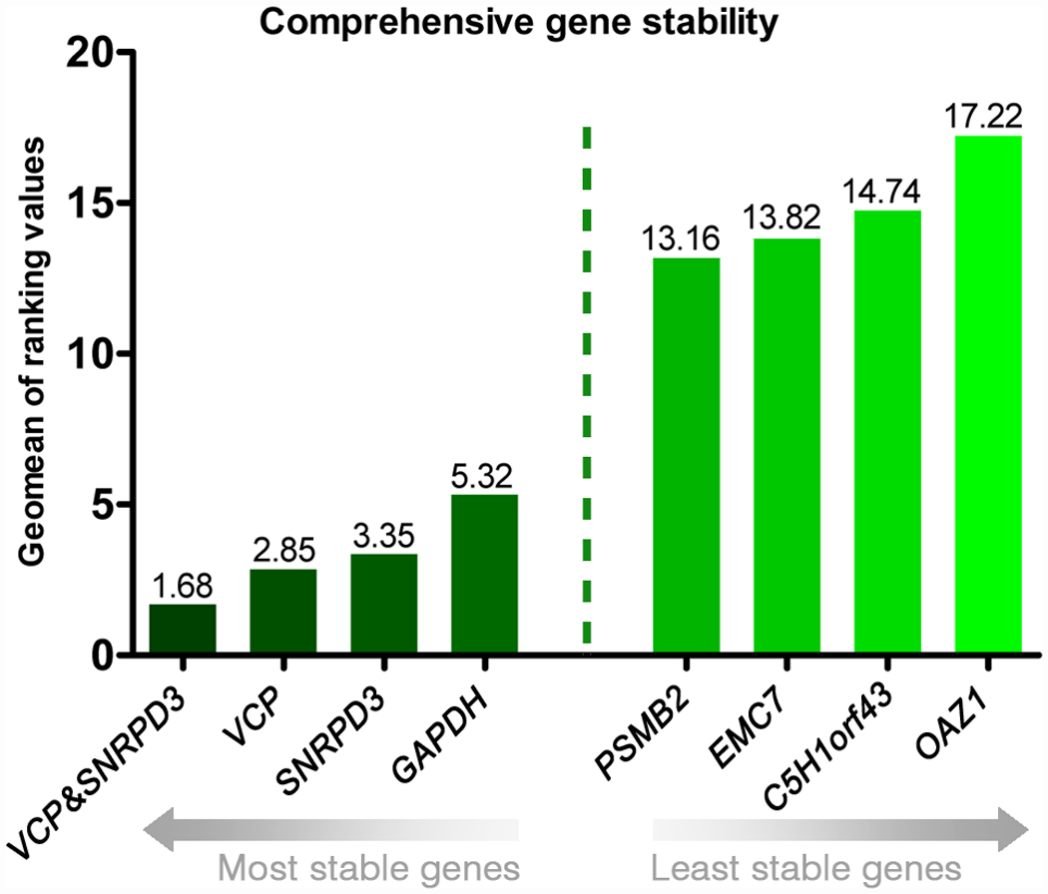
Expression uniformity analysis using the RefFinder software. The individual genes or the normalization factor calculated from the best-ranking, not co-expressed pair of genes are depicted. Only the “expressors” of highest and lowest ranks are presented.

Expression stability rankings produced by analytic algorithms like NormFinder and BestKeeper are often in overall agreement [27]. However, a discrepancy in stability rankings and outcomes between different analytical methods has been reported [24,25,48]. To increase the chance for deducing a “consensus” in the expression uniformity ranking of the novel candidate reference genes we used the comprehensive tool RefFinder (S5 File). The comprehensive approach identified *SNRPD3—*a core component of the spliceosome and *VCP*—an ATPase of the endoplasmic reticulum that prevents mutations caused by DNA damage, as the most stably expressed pair of genes in the biological context studied. Traditional RGs like *GAPDH*, *B2M*, *ACTB and UBB* were less uniformly expressed as documented by their ranks 3 and 12 to 14, respectively. If normalization should be performed with more than one RG, the pair *SNRPD3* and *VCP* is recommended for calculating a normalization factor (Fig 3).

**Fig 4:**
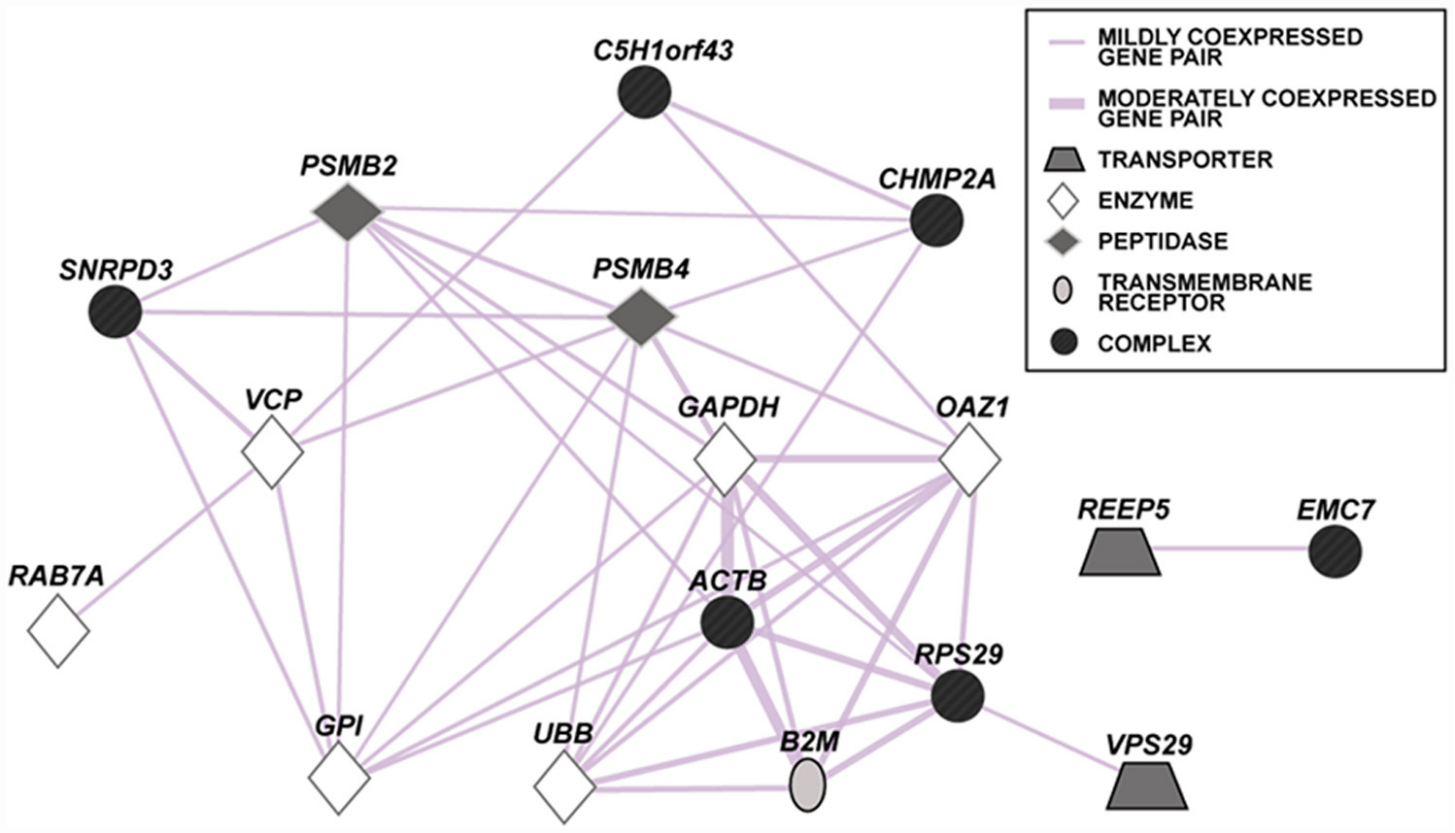
Co-expression analysis for all reference gene candidates according to human expression data contained in GeneMANIA. Two genes are linked if their expression levels are similar across conditions in a gene expression study, mostly derived from peer-reviewed publication data publicly accessible via the Gene Expression Omnibus (GEO). A thicker line indicates a higher co-expression. Note that *ERE-B* represents a repetitive element specific to the horse.

Finally, co-expression analysis of traditional and universal reference genes as well as the novel normalization candidates was performed using COEXPRESdb using all tissues available. For the best-performing gene pair, *VCP* and *SNRPD3*, absence of a significant co-expression was found (mutual rank: 716.7; Pearson’s coefficient: 0.33; S6 File). The latter values were far from being biologically significant (respective thresholds: < 30 [49] and > 0.55 [50]). While the Pearson’s correlation coefficient is affected by the function of the target gene and the method of gene expression database construction, the alternative use of correlation ranks helps to retrieve only genes that are functionally related [51]. Likewise, identification of co-expressed genes by GeneMANIA, containing peer-reviewed data collected mostly from the Gene Expression Omnibus (GEO), also failed to detect a co-expression for this pair of genes (Fig 4).

For seven genes (*C1orf43*, *CHMP2A*, *EMC7*, *GPI*, *RAB7A*, *VPS29* and *UBB*; S7 File) RT-qPCR primers could be designed based on a consensus sequence obtained by aligning the equine transcripts with all human orthologs available in the GenBank. The design of cross-species primers considerably reduces the likelihood of mismatches in their priming sites and hence improves quantification accuracy. For experimental validation of these seven RT-qPCR assays for human samples, RNA isolated from HeLa and Jurkat cells was used as template. The specificity of amplicons was demonstrated by agarose gel electrophoresis (S8 File).

## Conclusion

This study assayed the expression stability of highly conserved human exon orthologs in the neonatal horse ovary. Novel housekeeping genes identified in the related species *Homo sapiens* including the best-ranking gene pair of our study, *SNRPD3* and *VCP*, have been confirmed as more stable RGs for RT-qPCR profiling compared to traditional ones. The superiority of the novel RG set is characterized by high expression uniformity and obviously no co-expression. We attributed the successful application of the orthology clause in the next generation sequencing era to the extended number of transcripts identified, a resolution down to the level of individual exons and to the expression consensus obtained by profiling across a large set of target tissues.

The functional diversity of the novel orthologous RGs likely facilitates expression studies over a wide range of physiological and pathological contexts related to the neonatal equine ovary. However, for each biological context a specific subset of RGs needs to be validated from this extended pool of genes. With regard to context-specific RG validation in equine research, the ethical aspect needs to be strongly considered, as sample collection at different stages of development would involve inducing abortion in late pregnant mares or euthanizing young healthy foals.

In the near future RNA-seq analysis putatively performed for the neonatal and the adult equine ovary will help to further extend the repertoire of RGs for RT-qPCR normalization by adding context-optimized RGs with superior expression uniformity as shown before for the context of the mouse jejunum [34].

We also note an extension of the toolkit for human molecular biology by introducing novel validated RG assays for human samples that are in the truest sense in accordance with the MIQE guidelines regarding full transparency of oligonucleotide sequences [5].

## Supporting Information

S1 File The RT-qPCR assays for the novel reference genes detect all transcript isoforms currently known. (PDF)

S2 File RT-PCR product size of equine ovary sample determined on a 2% agarose gel stained with GelGreen. (EPS)

S3 File Estimated transcript abundance of all reference gene candidates. (XLSX)

S4 File Names and functions of reference gene candidates. (DOCX)

S5 File Complete list of expression uniformity analysis by the RefFinder software. (XLSX)

S6 File Co-expression analysis for all pairs of gene candidates using COEXPRESdb. (XLSX)

S7 File Validation of RT-qPCR assays for novel human reference genes. (XLSX)

S8 File RT-PCR product size of HeLa and Jurkat cells on a 2% agarose gel stained with GelGreen. (EPS)
