# Supplementary material for: The Orthology Clause in the Next Generation Sequencing Era: Novel Reference Genes Identified by RNA-seq in Humans Improve Normalization of Neonatal Equine Ovary RT-qPCR Data
Source: PLoS One. 2015 Nov 4;10(11):e0142122. doi: 10.1371/journal.pone.0142122 (PMC4633174; doi:10.1371/journal.pone.0142122)
Supplement: S1 File — (PDF) [file pone.0142122.s001.pdf]

File S1. The RT-qPCR assays for the novel reference genes detect all transcript isoforms currently known. The assay amplicon (red bars, labelled ”target“) and the transcript isoforms (black bars labelled with the respective accession numbers) were blasted against the horse genome (Equus caballus version 81.2) in the Ensembl Genome Browser [www.ensembl.org]. The genomic location of each gene is shown as dashed line. Protein coding regions are depicted as red boxes against green background.

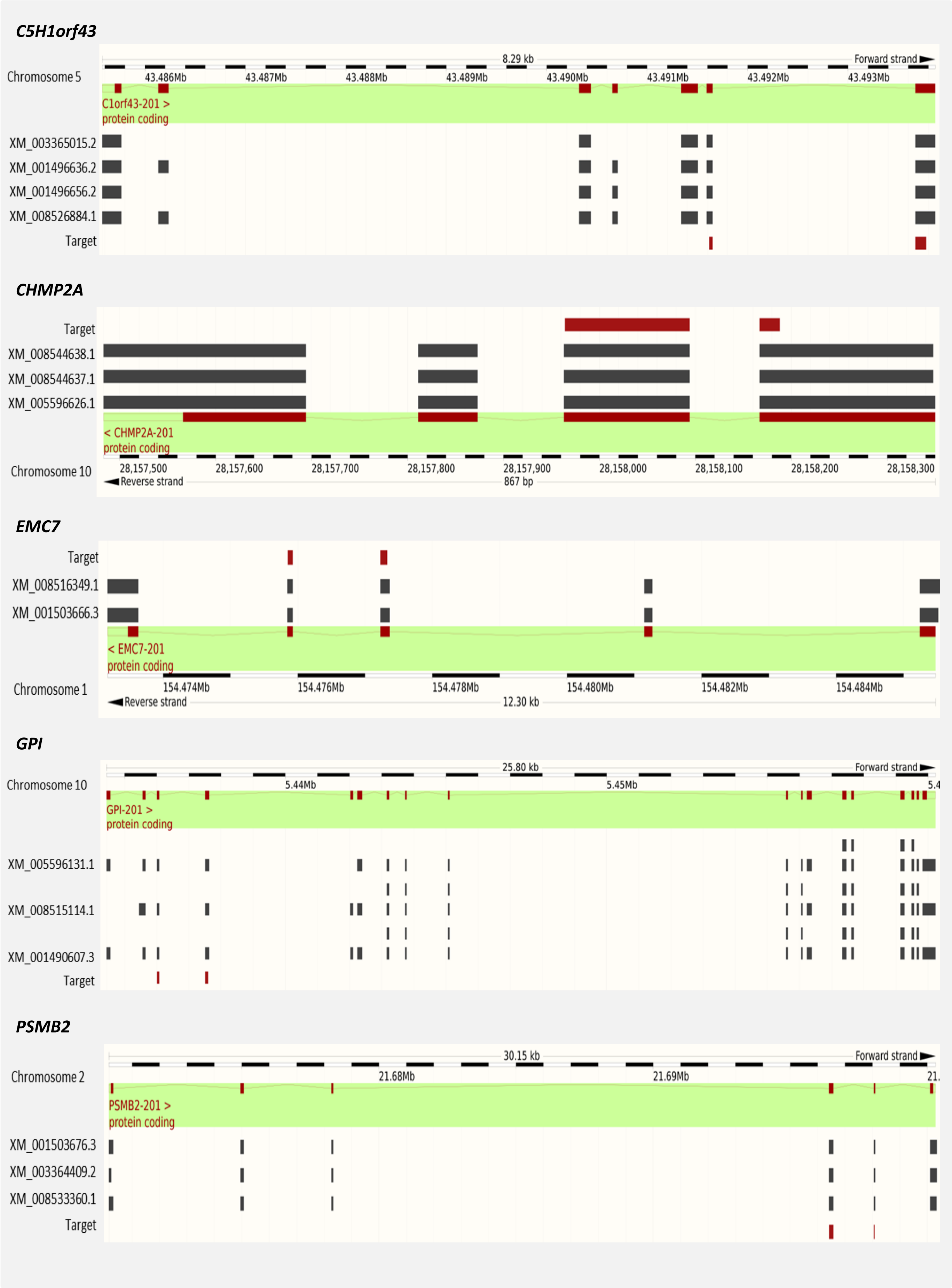

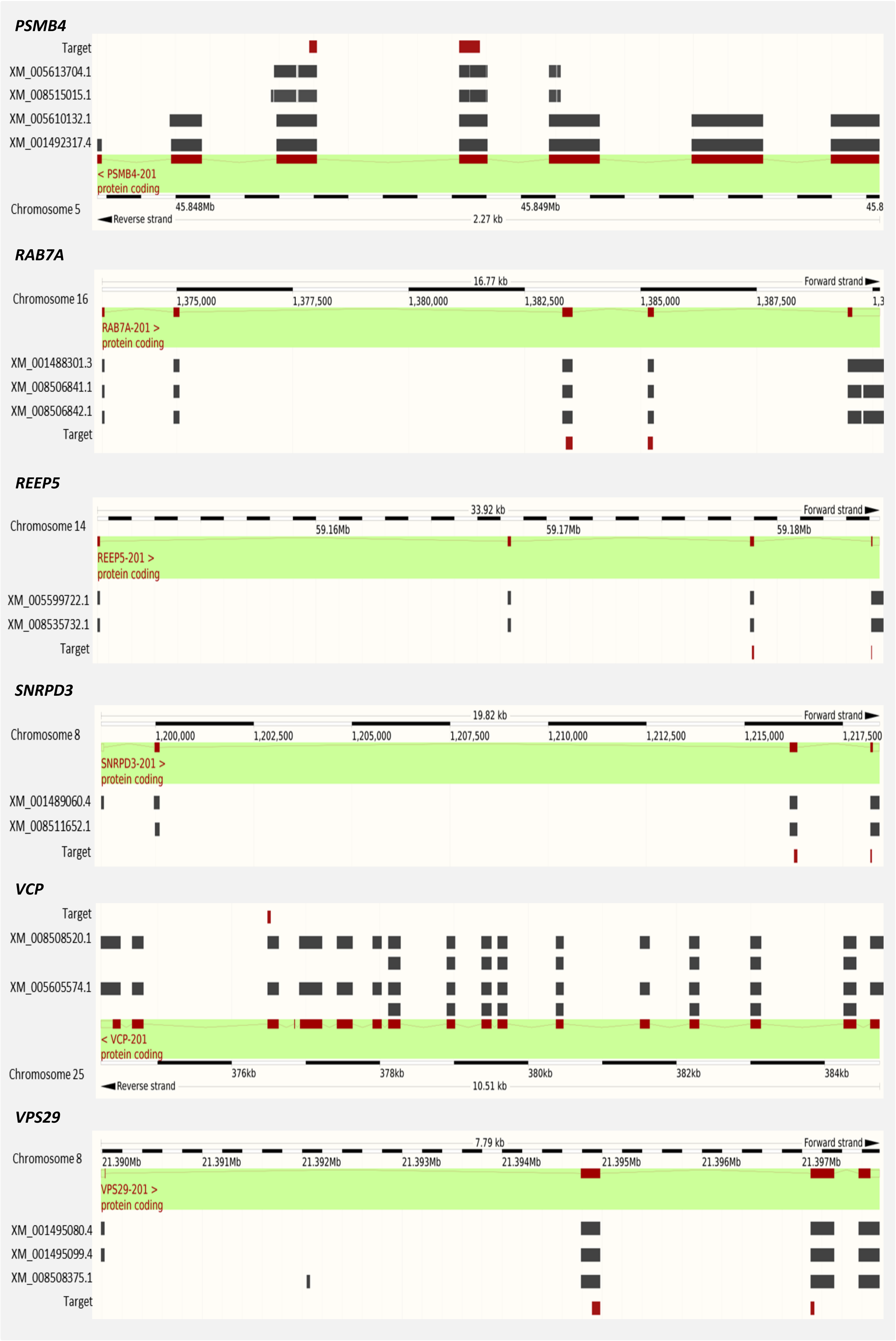
PSMB4
Target
XM_005613704.1
XM_008515015.1
XM_005610132.1
XM_001492317.4
< PSMB4-201
protein coding
Chromosome 5
45.848Mb
45.849Mb
◄Reverse strand
2.27 kb
RAB7A
16.77 kb
Forward strand ►
Chromosome 16
1,375,000
1,377,500
1,380,000
1,382,500
1,385,000
1,387,500
RAB7A-201 >
protein coding
XM_001488301.3
XM_008506841.1
XM_008506842.1
Target
REEP5
33.92 kb
Forward strand ►
Chromosome 14
59.16Mb
59.17Mb
59.18Mb
REEP5-201 >
protein coding
XM_005599722.1
XM_008535732.1
Target
SNRPD3
19.82 kb
Forward strand ►
Chromosome 8
1,200,000
1,202,500
1,205,000
1,207,500
1,210,000
1,212,500
1,215,000
1,217,500
SNRPD3-201 >
protein coding
XM_001489060.4
XM_008511652.1
Target
VCP
Target
XM_008508520.1
XM_005605574.1
< VCP-201
protein coding
Chromosome 25
376kb
378kb
380kb
382kb
384kb
◄Reverse strand
10.51 kb
VPS29
7.79 kb
Forward strand ►
Chromosome 8
21.390Mb
21.391Mb
21.392Mb
21.393Mb
21.394Mb
21.395Mb
21.396Mb
21.397Mb
VPS29-201 >
protein coding
XM_001495080.4
XM_001495099.4
XM_008508375.1
Target
