# Supplementary material for: The Orthology Clause in the Next Generation Sequencing Era: Novel Reference Genes Identified by RNA-seq in Humans Improve Normalization of Neonatal Equine Ovary RT-qPCR Data
Source: PLoS One. 2015 Nov 4;10(11):e0142122. doi: 10.1371/journal.pone.0142122 (PMC4633174; doi:10.1371/journal.pone.0142122)
Supplement: S4 File — (DOCX) [file pone.0142122.s004.docx]

**File S4. Names and functions of reference gene candidates**

| Gene symbol | RG Category | Gene name | Gene function |
| --- | --- | --- | --- |
| *C5H1orf43* | novel | chromosome 5 open reading frame, human *C1orf43* | coenzyme binding |
| *CHMP2A* | novel | charged multivesicular body protein 2A | protein transport |
| *EMC7* | novel | ER membrane protein complex subunit 7 | member of ER membrane protein complex |
| *GPI* | novel | glucose-6-phosphate isomerase | member of glucose phosphate isomerase protein family |
| *PSMB2* | novel | proteasome subunit, beta type, 2 | [proteasome subunit](http://www.ebi.ac.uk/QuickGO/GTerm?id=GO:0001530) with [threonine-type endopeptidase activity](http://www.ebi.ac.uk/QuickGO/GTerm?id=GO:0004298) |
| *PSMB4* | novel | proteasome subunit, beta type, 4 | [proteasome subunit](http://www.ebi.ac.uk/QuickGO/GTerm?id=GO:0001530) |
| *RAB7A* | novel | member RAS oncogene family | member of RAS oncogene family |
| *REEP5* | novel | receptor accessory protein 5 | promotes functional cell surface expression of olfactory receptors |
| *SNRPD3* | novel | small nuclear ribonucleoprotein D3 | poly(A) RNA binding |
| *VCP* | novel | valosin containing protein | component of the proteasome complex  ATPase activity  poly(A) RNA binding |
| *VPS29* | novel | vacuolar protein sorting 29 homolog | subunit of retromer complex |
| *ACTB* | traditional | actin, beta | structural constituent of cytoskeleton |
| *GAPDH* | traditional | glyceraldehyde-3-phosphate dehydrogenase | organization and assembly of cytoskeleton |
| *UBB* | traditional | ubiquitin B | DNA repair |
| *B2M* | traditional | beta-2-microglobulin | component of class I major histocompatibility complex |
| *OAZ1* | universal | ornithine decarboxylase antizyme 1 | regulation of polyamine biosynthesis |
| *RPS29* | universal | ribosomal protein S29 | [structural constituent of ribosome](http://www.ebi.ac.uk/QuickGO/GTerm?id=GO:0003735) |
| *ERE-B* | repeat element | equine repetitive elements B | short interspersed nuclear element |
| *RN18S* | ribosomal | 18S ribosomal RNA | [poly(A) RNA binding](http://www.ebi.ac.uk/QuickGO/GTerm?id=GO:0044822)  [methyltransferase activity](http://www.ebi.ac.uk/QuickGO/GTerm?id=GO:0008168) |
| *RN28S* | ribosomal | 28S ribosomal RNA | [poly(A) RNA binding](http://www.ebi.ac.uk/QuickGO/GTerm?id=GO:0044822)  [methyltransferase activity](http://www.ebi.ac.uk/QuickGO/GTerm?id=GO:0008168) |
